# Femoral artery remodeling after aerobic exercise training without weight loss in women

**DOI:** 10.1186/1476-5918-7-13

**Published:** 2008-09-08

**Authors:** Manning J Sabatier, Earl H Schwark, Richard Lewis, Gloria Sloan, Joseph Cannon, Kevin McCully

**Affiliations:** 1 The University of Georgia, Department of Kinesiology, Athens, GA 30602, USA; 2 The University of Georgia, Department of Foods and Nutrition, Athens, GA 30603, USA; 3 Medical College of Georgia, Department of Biomedical and Radiological Technologies, Augusta, GA 30912, USA; 4 Health & Fitness Management, Clayton State University, 2000 Clayton State Blvd, Morrow, GA 30260, USA

## Abstract

**Background:**

It is currently unclear whether reductions in adiposity mediate the improvements in vascular health that occur with aerobic exercise. The purpose of this longitudinal study of 13 healthy women (33 ± 4 years old) was to determine whether 14 weeks of aerobic exercise would alter functional measures of vascular health, namely resting aortic pulse wave velocity (aPWV, an index of arterial stiffness), femoral artery diameter (D_FA_), and femoral artery blood flow (BF_FA_) independent of changes in adiposity.

**Methods:**

Aerobic fitness was assessed as VO_2_peak normalized to fat-free mass, and adiposity (percent body fat) was determined by dual energy x-ray absorptiometry. Serum concentrations of proteins associated with risk for cardiovascular disease, including C-reactive protein (CRP), soluble intercellular adhesion molecule-1 (sICAM-1), and leptin, were also measured. Subjects cycled for 50 minutes, 3 times per week.

**Results:**

Aerobic fitness normalized to fat-free mass increased 6% (P = 0.03) whereas adiposity did not change. Resting D_FA _increased 12% (P < 0.001) and resting shear rate decreased 28% (P = 0.007). Aortic PWV, and serum sICAM-1, CRP and leptin did not change with training.

**Conclusion:**

Significant reductions in adiposity were not necessary for aerobic exercise training to bring about improvements in aerobic fitness and arterial remodeling. Peripheral arterial remodeling occurred without changes in central arterial stiffness or markers of inflammation.

## Background

Low aerobic fitness is a significant and prevalent risk factor for future cardiovascular disease (CVD) [1,2]. An increase in aerobic fitness through exercise training reduces CVD risk [3,4]. In keeping with this concept, increased fitness has been shown to improve functional measures of cardiovascular health, such as aortic stiffness measured using aortic pulse wave velocity (aPWV) [5]. It also results in femoral arterial remodeling as determined from Doppler ultrasound imaging of arterial diameter [6-8]. In addition, elevated resting shear stress in the femoral artery results from disuse of the lower extremities [9,10]. Considering that disuse is also associated with increased CVD (reviewed in [11]), elevated resting shear stress may also be associated with increased CVD risk.

Elevated adiposity also increases the risk for CVD and has increased in prevalence over the last several decades [12-14]. Therefore, reducing the prevalence of obesity is a major public health initiative. Elevated adiposity is also associated with low physical activity and weight reduction typically involves an increase in physical activity. It is not presently clear whether elevated adiposity is an independent risk factor for CVD or merely a proxy for low physical activity, low fitness, and poor diet. Prospective studies have shown that aerobic fitness reduces the negative effect of adipose tissue on CVD mortality [15]. Additionally, weight loss programs have been associated with repetitive weight loss and weight gain, net increases in weight and increased CVD mortality [16-18]. This suggests that programs designed to improve cardiovascular health may do better to focus on improved aerobic fitness rather than weight loss.

The circulating concentrations of several proteins that may originate from, or influence the development of, atherosclerotic plaques are currently under investigation as adjuncts to cholesterol/lipoprotein measurements in assessment of risk for cardiovascular disease. Among those reported to be independent predictors of cardiovascular disease risk are C-reactive protein (CRP) [19], soluble intercellular adhesion molecule-1 (sICAM-1) [20], and leptin [21].

Elevated levels of CRP and sICAM-1 have been associated with CVD [22], as well as with adiposity [23,24]. Adiposity is also associated with elevations in leptin, an adipocyte-derived, 16 kDa hormone/cytokine that influences energy balance, thermoregulation and reproductive function [25]. Leptin receptors have been identified on vascular cells [26], and several in vitro and animal studies have indicated that leptin may influence atherosclerotic plaque development (reviewed in [27]). A reduction in these biological proteins might confer protection against the progression of atherosclerosis.

Previous studies that have evaluated the interaction between aerobic fitness and adiposity have commonly used body mass index (BMI) as an index of adiposity. BMI is not a measure of the amount of adipose tissue an individual has and its relationship to adiposity, or percent body fat, may vary considerably between individuals [28]. Furthermore, without an accurate measure of adiposity, maximal oxygen consumption as an index of aerobic fitness is compromised. For example, total body weight is typically used to normalize oxygen consumption. Therefore, increased adiposity will automatically lower aerobic fitness irrespective of the maximal capacity of the individual's cardiorespiratory system and muscles to deliver and utilize oxygen. Dual energy x-ray absorptiometry (DXA) yields accurate estimates of fat-free mass [29] which can be used to normalize maximal oxygen consumption and help minimize this limitation [30]. The purpose of this study was to determine whether a 14-week aerobic exercise program would 1) improve aerobic fitness without changing adiposity, 2) improve functional measures of vascular health, and 3) reduce serum concentrations of proteins associated with risk for cardiovascular disease. A secondary purpose was to determine whether changes in functional measures of vascular health and serum concentrations of proteins associated with risk for cardiovascular disease were dependent upon changes in aerobic fitness.

## Methods

### Subjects

Thirteen sedentary healthy women (33 ± 4 years old) were studied on 2 days within a 7-day span, before and after a 14-week cycle training program. Blood samples were taken and physiological measurements other than cardiorespiratory fitness were made during the morning after an overnight fast before and after the training intervention for all subjects. Cardiorespiratory fitness was assessed on a separate day from other measurements. Subjects abstained from caffeine or alcohol in the 12 hours prior to physiological measurements. Subjects were screened to exclude for self-reported cardiovascular disease, neuromuscular disease, hypertension, diabetes, and smoking. All procedures were approved by the Human Assurance Committee at the Medical College of Georgia and the Institutional Review Board at The University of Georgia and conformed to the regulations laid out in the declaration of Helsinki on the use of human subjects in research. All subjects were instructed as to the procedures involved in the project and gave written informed consent before undergoing testing procedures.

### Cardiorespiratory fitness

Maximal exercise testing was performed using a friction-braked Monark cycle ergometer as described previously [31]. Although maximal oxygen consumption measured using the stationary cycle is on the order of 10% lower than that generated from performance of a treadmill graded exercise test [32], the stationary cycle was used to avoid the possible effects of larger body sizes on maximal testing with protocols involving ambulation. The test began with 4 minutes of cycling without resistance followed by an increase of 15 W every minute [31]. Oxygen consumption was calculated based on measures of pulmonary ventilation and mixed expired oxygen and carbon dioxide using the Vmax Spectra (Yorba Linda, CA). Heart rate (EKG) and blood pressure (auscultation) were assessed during each stage and subjects provided subjective ratings of perceived exertion during every other stage of the test. Peak oxygen consumption (VO_2_peak) was taken as the maximal 30-second average and normalized to fat-free mass [30].

### Body composition

Body composition [total bone mineral content (BMC; kg), fat mass (FM; kg), fat-free soft tissue mass (FFST; kg), and % body fat] was measured at baseline and follow-up using DXA (Delphi A; S/N 70467; Hologic Inc., Bedford, MA). The same technician analyzed all baseline and follow-up scans using Hologic Whole Body Analysis software, version 11.2.

Quality assurance for DXA was carried out by daily calibration against the manufacturer's standard phantom (Hologic anthropomorphic spine phantom, model DPA/QDR-1; SN 9374). Fat Mass and FFST measures were calibrated by concurrently scanning (with each total body scan) an external three-step soft tissue wedge (Hologic, Inc) composed of different thickness levels of aluminum and lucite, calibrated against stearic acid (100% fat) and water (8.6% fat). Test-retest measurements using DXA in premenopausal women (n = 10) scanned twice in our lab during a 7-day period demonstrated CVs for total body BMC of 1.2% and percent fat of 2.0%.

### Laboratory measurements

Blood samples were obtained by venipuncture after a 12-hour fast to assay for CRP, ICAM-1, and leptin. Serum was stored in a -70°C freezer for later analysis. Serum CRP, leptin and ICAM-1 were assayed using ELISAs constructed with separately-purchased reagents. Primary antibodies were polyclonal rabbit anti-human C-reactive protein (A0073, DAKO, Carpintera, CA), monoclonal mouse anti-human leptin (MAB398, R, & D Systems, Minneapolis, MN), and monoclonal mouse anti-human ICAM-1 (MAB720, R & D Systems). For all assays, capture antibodies were coated on 96-well polystyrene plates (Corning #3590, Corning, NY). Samples and standards were incubated in duplicate in plates overnight at 4°C. Detection limits, inter- and intra-assay variability's for each assay were: CRP – 0.3 ng/ml, 12%, 3%; leptin – 0.06 ng/ml, 5%, 2%; ICAM-1 – 0.14 ng/ml, 9%, 6%.

To determine if menstrual status influenced any of the measured variables, serum estradiol and progesterone concentrations were measured using ELISA kits from Diagnostic Systems Labs (Webster, TX). The estradiol assay (DSL-10-4300) had a sensitivity of 7 pg/ml with intra- and inter-assay variability's of 5 and 8%, respectively. The progesterone assay (DSL-10-3900) had a sensitivity of 0.13 ng/ml with intra- and inter-assay variability's of 7 and 5%, respectively.

### Aortic pulse wave velocity and arterial blood pressure

Aortic pulse wave velocity was used to evaluate central arterial stiffness [33] using the Biopac MP100 physiological data acquisition system (Biopac Systems Inc., Goleta, CA) and AcqKnowledge software (v3.7.3). One electrode was placed over the superior and another over the distal-most boundary of each scapulae, with a third placed on the lower back. A 40.6–66.0 cm Biopac blood pressure cuff (RX120F) was interfaced with a TSD120 transducer and DA100C general purpose transducer amplifier. All recordings were made after 30 minutes of supine rest. Peripheral arterial blood pressure was measured during this period over the brachial artery of the left arm with a semi-automated blood pressure machine (Datascope, Mahwah, NJ) using an appropriate-sized cuff [34]. The average of 3 to 4 blood pressure readings was used for subsequent analysis.

Distance from the sternal notch to the thigh cuff was measured with a standard tape measure maintaining a parallel orientation of the tape measure to the examination table without conforming to body topography. The time difference between the inflection point of the systolic pulse wave and the peak of the R-wave of the EKG signal was calculated for 30 cardiac cycles for which the pulse wave inflection point was most distinct. A time constant of 0.05 sec was subtracted from this difference to correct for isovolumic contraction phase that precedes ejection [35]. The calculation was performed as follows: aPWV = distance (cm) ÷ [time difference (sec) - 0.05 (sec)]. Day-to-day reproducibility with 2 testing days was evaluated in 17 subjects (11 females, 6 males; 22 ± 2 yrs of age) using this method of evaluating aPWV with an average day-to-day difference of 8.1%.

### Femoral artery diameter, blood flow, and shear rate

In all tests the subject rested quietly in the supine position for 15 minutes before data acquisition began. One sonographer performed all tests for this study. B-mode imaging was used to visualize the artery 1 – 5 cm distal to the femoral bifurcation using a LogiQ 400CL (General Electric, Rainbow City, AL) with a 7–13 MHz linear-array ultrasound transducer. B-mode images were recorded during diastole for off-line diameter (mm) measurements. Resting blood velocity (cm/second) was assessed using pulsed Doppler ultrasound recorded in the longitudinal view using an insonation angle between 45° and 60°. The velocity gate was set to include the entire lumen area. Time averaged maximum velocity (TAMAX) was auto calculated every cardiac cycle by the GE 400CL advanced vascular program. TAMAX values were acquired and saved directly to a computer using specially coded optical character recognition software (NI LabVIEW 6i, Austin, TX), allowing data acquisition on a beat-by-beat basis. Image files were opened using semi-automated software specially coded for use with NI LabVIEW. Arterial diameter was measured manually by applying straight lines conforming to the wall-lumen interface of two-dimensional images captured during data collection. Blood flow (mL/min) is the product of blood velocity and artery cross-sectional area and was calculated as follows: blood flow = [π(diameter/2)^2^]*[velocity*60 seconds/minute]. We used shear rate as an estimate of shear stress. Shear rate was calculated as blood velocity ÷ diameter [36].

Day-to-day reproducibility for resting diameter, blood velocity, blood flow, and shear rate was evaluated between 2 testing days in 15 subjects (9 females, 6 males; 21 ± 1 yrs of age) that were not part of the training study. Average day-to-day difference for diameter, blood velocity, blood flow, and shear rate was 1.7%, 30.0%, 32.1%, and 19.9%, respectively.

### Training intervention

The training program was conducted at the University of Georgia's Adult Fitness Center. Subjects were scheduled to exercise three times per week for 14 weeks under direct supervision of one of the study's investigators but were permitted to conduct exercise sessions on their own on an infrequent basis in the event of extenuating personal circumstances. Any subject who utilized this option had to: 1) have access to a stationary cycle, 2) be thoroughly familiar with the protocol, and 3) be proficient at monitoring heart rate while cycling. This was infrequent for 11 of the 13 subjects. Although two subjects conducted 50% of their exercise sessions away from our direct supervision, their results were not markedly different from the rest of the group.

**Figure 1:**
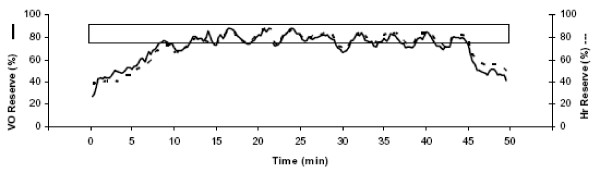
**Heart rate and oxygen consumption during one training session for one subject.** Heart rate and oxygen consumption, as percentages of reserve, changed concurrently throughout the session and both remained above 60% for the entire 40-minute body of the training session. The upper and lower edge of the box super-imposed on the graph corresponds with 90% and 75% of HR reserve, respectively.

Airdyne bikes were utilized for training with minimal or no use of handles. Cycling was performed at a comfortable, self-chosen pace during the initial 3–4 sessions of the training program. After this induction phase, ten 2-minute alternating intensity bouts (high and low) followed the warm-up. Each session included 5 minutes of warm up, 40 minutes of intermittent intensity cycle training (2 minutes high, 2 minutes low), and 5 minutes of cool down, for a total of 50 minutes of pedaling. High intensities were chosen to elicit a heart rate of 75–90% of heart rate reserve and were achieved through an increase in pedaling speed. Low intensities were chosen to elicit 55–65% of heart rate reserve. Heart rate responses were monitored to ensure that proper intensities were achieved. Figure 1 illustrates results of one session for one subject while heart rate and expiratory gases were measured using EKG and open circuit spirometry. Percent heart rate reserve and percent VO_2 _reserve were closely matched during the training session (r = 0.92), supporting our use of heart rate to control and monitor training intensity.

### Statistical analysis

Statistical analyses were performed with the SPSS (version 13.0) statistical package. Measurements made before and after training were evaluated using paired Student's t-tests. Non-normal data were logarithmically transformed and verified as normal before proceeding with parametric analysis. Results are reported as mean (SD) unless otherwise noted and statistical significance was determined at P ≤ 0.05.

## Results

The physical characteristics of the subjects are shown in Table 1. The training program did not result in a change in body weight or BMI. There was a trend for small increases in fat-free mass and decreases in fat mass, but these changes were not statistically significant. Training resulted in a significant decrease in resting heart rate (4.6%), although systolic and diastolic arterial blood pressure did not change.

**Table 1:** Physical characteristics before and after training.

	Pre Mean (SD)	Post Mean (SD)	P-value
Age (yr)	33 (4)	---	---
Height (cm)	160 (6)	---	---
Weight (kg)	74.4 (21.6)	74.3 (21.4)	0.43
BMI (kg/m^2^)	29.1 (9.1)	29.1 (9.0)	0.38
Body fat (%)	37.3 (7.3)	36.5 (7.0)	0.08
Fat mass (kg)	29.0 (13.8)	28.3 (13.8)	0.12
Fat-free mass (kg)	45.4 (8.4)	46.0 (8.2)	0.08
			
Heart Rate (bpm)	65 (9)	62 (9)	0.01
Systolic BP (mmHg)	110 (9)	108 (11)	0.14
Diastolic BP (mmHg)	70 (7)	68 (7)	0.10

Data in Pre and Post columns are shown as mean (SD).

P-values were calculated based on paired Student's t-tests.

VO_2_peak increased 6% with training (44.5 ± 6.8 vs. 47.1 ± 5.7 ml·kgFFM^-1^·min^-1^, P = 0.03). In addition, at the highest work level of the maximal exercise test that all subjects were able to perform before and after training (115 W), there was a decrease in heart rate after training that approached statistical significance (155 vs 150 bpm, P = 0.07), and a significant reduction in the rate pressure product (2.5·10^4 ^vs 2.2·10^4 ^mmHg*bpm, P = 0.02). These changes are consistent with an improvement in aerobic fitness.

**Figure 2:**
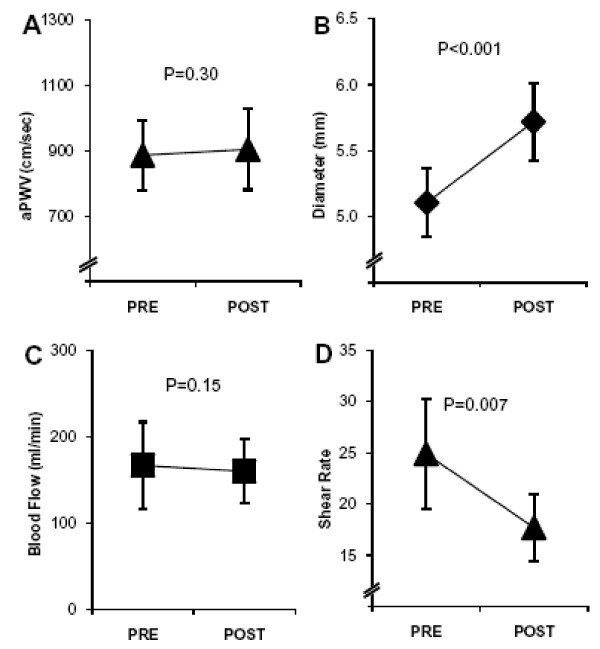
**aPWV (A) did not change with training.** Femoral artery diameter (B) increased 12%, femoral artery blood flow (C) did not change, and femoral artery shear rate (D) decreased 29% with training. Data are illustrated as mean ± 95% C.I.

Training resulted in a significant increase in femoral artery diameter from 5.1 ± 0.5 to 5.7 ± 0.5 mm (P < 0.001), whereas femoral artery blood flow did not change (167 ± 89 vs. 160 ± 68, P = 0.15) (Figure 2). In line with these two findings, there was a significant decrease in calculated shear rate from 24.9 ± 9.5 to 17.6 ± 6.1 (P = 0.007). aPWV did not change with training (886 ± 196 vs. 903 ± 227, P = 0.30).

No significant changes in CRP, sICAM or leptin were observed with the training program (Table 2). Leptin values did correlate with adiposity (r = 0.81 pre, and 0.82 post). No significant differences in serum estradiol or progesterone were observed in the pre- versus post-training samples.

## Discussion

The key finding of this study was that femoral artery diameter increased with an aerobic training program that resulted in an increase in fitness without a decrease in adiposity in middle aged women. The exercise program used in this study was similar to commonly used aerobic training programs in that the exercise intensity was within the guidelines suggested by the American College of Sports Medicine [37]. What was slightly different was our use of varying exercise intensities within each training session in order to provide variety and to reduce boredom for participants. The participants overwhelmingly viewed the training paradigm favorably and indicated that the program had achieved this goal. VO_2_peak values increased 10% when normalized to body weight. When normalized to lean tissue, the increase in VO_2_peak was 6%, which is slightly less than reported in a comparable study that also normalized VO_2_peak to lean body mass [38]. This may be due to a shorter training program in the present study, as well as subtle differences in the composition of exercise sessions between the two studies. We had similar rates of adherence to the training program. The improvements in aerobic fitness, in combination with the reduction in rate-pressure product during maximal exercise testing, suggest a significant overall physiological adaptation.

The training program implemented in this study resulted in femoral artery remodeling that was independent of adiposity. Physical activity [6,39] and aerobic fitness [40] have previously been found to be associated with larger femoral artery diameters. Although previous studies have found that aging and/or long-term atherosclerotic plaque accumulation also result in increased artery size as well as central arterial stiffness [41], the current study evaluated subjects over a period of only 16 weeks and the study population was homogeneous in age and free of cardiovascular disease. Our findings of increased aerobic fitness and decreased heart rate also suggest that sympathetic drive was reduced. One might expect that a reduction in sympathetic outflow to the legs would result in increased dilation and consequently an increase in artery diameter. However, previous data suggest that endurance-training results in either no or slightly elevated sympathetic drive to the leg at rest [42], which would have resulted in an opposite change in diameter. Therefore, it is more likely that the increase in femoral artery diameter found in this cohort is a sign of remodeling than of arterial deterioration or increased dilation.

The increase in arterial diameter was associated with a decrease in resting femoral artery shear rate. Low resting shear rates represent a positive adaptation to exercise training as elevated shear rates during exercise induce the positive arterial adaptations [43-45]. These adaptations result in larger arteries that subsequently have reduced shear rates at rest. Adults with spinal cord injury are unable to increase shear rates in their affected limbs and subsequently have smaller arteries and elevated resting shear rates compared to able-bodied control groups [9,10]. This suggests that increased resting shear rate is at least associated with vascular deterioration since spinal cord injury also results in accelerated CVD progression [46]. Therefore, our results can be cautiously interpreted to suggest that the training program improved vascular health of the study participants.

Exercise training did not decrease arterial stiffness (i.e., aPWV) in this study. Although cross-sectional studies suggest that higher fitness is associated with lower aPWV, age may help explain our results. Aortic PWV was found to increase with age in a group of 480 Chinese citizens (ages 3 to 89) in whom atherosclerosis is known to be rare and who did not have CVD [47]. Also, cross-sectional studies of endurance trained older adults do not find the age-related increases in aPWV found in sedentary older adults [48,49]. These data suggest that age is a significant and independent predictor of central arterial stiffness and that its effects are lessened when accompanied by years of aerobic exercise. Therefore, central arterial stiffness may have not been sufficiently elevated in this study for exercise and increased fitness to evoke a measurable reduction in this study. Alternatively, the morphological aspects of the aorta that aPWV reflects, namely atherosclerotic plaque accumulation, loss of elastin, and collagen build-up, may not be significantly reversible in the time frame of this study [50].

Aerobic training did not reduce serum sICAM-1 concentrations. This result was contrary to our hypothesis that training-induced reductions in shear stress would reduce sICAM-1 levels. This prediction was based on evidence that shear stress increases ICAM-1 expression and release of sICAM-1 from isolated human saphenous vein endothelial cells [51]. However other factors, including paracrine influences of IL-1β and TNFα can also increase ICAM-1 expression and shedding by endothelial cells [52]. A year-long study of weight reduction through exercise and diet reported a significant 26% reduction in serum ICAM-1 [24], whereas a 12-week aerobic training program with weight maintenance reported no changes in serum ICAM-1 [53].

Aerobic training did not affect serum CRP concentrations. In fact, the intrasubject coefficient of variability in the present study (36%) was less than that reported in other studies which had no intervention of any kind (42–61%, reviewed in [54]). A previous study reported reductions in serum CRP in middle-aged women after a 2-year weight loss program that coupled reduced caloric intake with increased physical activity [55]. In contrast, serum CRP did not change in 9–15 year old girls after a 12-week aerobic training program involving a weight-maintaining diet [53]. Other studies have found a positive relationship between CRP and adiposity [24,56]. Taken together, these studies indicate that CRP covaries with adiposity, but not aerobic fitness.

Consistent with previous studies [57,58], we also found that leptin levels correlated with adiposity. While one study involving a very low energy diet and weight loss detected an independent effect of exercise training on leptin concentrations by partial regression analysis [58], our results were in agreement with a study reporting that exercise without fat loss has little effect on leptin [57]. We found no evidence that exercise training and increased aerobic fitness lowered leptin concentrations independent of adiposity.

This study had some limitations that may limit interpretation of the results. For example, a potential criticism is the lack of a control group. Traditional cross-sectional studies use both experimental and control groups to examine the effect of the intervention or exposure. However, this study used a longitudinal, repeated measures design where the pre-treatment measures from the experimental group serve as the control. In the repeated measures design all the participants serve as their own control because they are involved in the experimental and control groups. We did not employ a time-matched control group that had measurements taken twelve weeks apart. Instead, each subject's pre-treatment data served as control data since the pre and post data represent measures of the same physiological variable, except without a preceding training period. This design is ideal when a limited number of participants are available because it increases the statistical power relative to sample size, and therefore the efficiency and sensitivity of the experiment [59,60]. In addition, we tested a small cohort without signs of cardiovascular disease. Therefore, we feel that the limitations of this study potentially result from the ability to generalize to diseased populations rather than from the lack of a control group. Although the results of this study should be interpreted with caution, it has provided salient findings using precise measurements that will assist in the design of future studies that are larger in breadth.

## Conclusion

In conclusion, this study found that 14 weeks of aerobic exercise increased aerobic fitness and favorably altered resting femoral artery diameter and shear rate in middle-aged women without changing adiposity. Future studies seeking to exercise-train sedentary populations may do well to use variable intensity programs to increase subjects' interest and potentially increase adherence and retention. The interpretations of this study are inherently limited because long-term follow-up would be required to ultimately determine how these findings relate to progression of CVD. However, these results suggest that aerobic training positively influences functional measures of arterial health without a concomitant loss of body fat but that circulating levels of biological proteins associated with CVD risk may be more strongly linked to adiposity than to fitness and/or require longer exercise interventions.

## Competing interests

The authors declare that they have no competing interests.

## Authors' contributions

MJS conceived of the study, conducted and coordinated the experiments, performed data analysis, and drafted the manuscript. EHS participated in preparation of the study design, conducted experiments and exercise training, and participated in writing the manuscript. RL participated in the preparation of the study design and in writing the manuscript. GS maintained regulatory compliance and performed immunoassay of serum samples, and performed data analysis. JC maintained regulatory compliance, administered the budget, and participated in data analysis and writing sections of the manuscript. KM conceived of the study and participated in its design and coordination, and in writing the manuscript. All authors read and approved the final manuscript.

**Table 2:** Serum proteins associated with CVD, before and after training.

	Pre Median (interquartile range) or Mean (SD)	Post Median (interquartile range) or Mean (SD)	P-value
CRP (mg/L) †	2.07 (0.86, 3.07)	1.77 (0.38, 2.46)	0.18
sICAM-1 (ng/mL)	169 (37)	172 (74)	0.40
Leptin (ng/mL)	30.7 (22)	26.8 (20)	0.07

† Mean (interquartile range) for the CRP data and Mean ± SD for the other data.

P-values were calculated based on paired Student's t-tests.
